# Hepatic Osteodystrophy: The Mechanism of Bone Loss in Hepatocellular Disease and the Effects of Pamidronate Treatment

**DOI:** 10.6061/clinics/2017(04)07

**Published:** 2017-04

**Authors:** Adriano L. Spirlandeli, Ingrid Dick-de-Paula, Ariane Zamarioli, Vanda Jorgetti, Leandra N.Z. Ramalho, Marcello H. Nogueira-Barbosa, Jose B. Volpon, Alceu A. Jordão, Fernando Q. Cunha, Sandra Y. Fukada, Francisco J.A. de Paula

**Affiliations:** I Departamento de Medicina Interna, Faculdade de Medicina de Ribeirão Preto, Universidade de São Paulo, Ribeirão Preto, SP, BR; II Departamento de Biomecânica, Medicina e Rehabilitação do Aparelho Locomotor, Faculdade de Medicina de Ribeirão Preto, Ribeirão Preto, SP, BR; III Departamento de Nefrologia, Faculdade de Medicina, Universidade de São Paulo, São Paulo, SP, BR; IV Departamento de Patologia, Faculdade de Medicina de Ribeirão Preto, Universidade de São Paulo, Ribeirão Preto, SP, BR; V Departamento de Farmacologia, Faculdade de Medicina de Ribeirão Preto, Universidade de São Paulo, Ribeirão Preto, SP, BR; VI Departamento de Física e Química, Faculdade de Ciências Farmacêuticas de Ribeirão Preto, Universidade de São Paulo, Ribeirão Preto, SP, BR

**Keywords:** Hepatic Osteodystrophy, Osteoporosis, Mice, Bone Remodeling, Ccirrhosis

## Abstract

**OBJECTIVES:**

The present study was designed to evaluate the bone phenotypes and mechanisms involved in bone disorders associated with hepatic osteodystrophy. Hepatocellular disease was induced by carbon tetrachloride (CCl_4_). In addition, the effects of disodium pamidronate on bone tissue were evaluated.

**METHODS:**

The study included 4 groups of 15 mice: a) C = mice subjected to vehicle injections; b) C+P = mice subjected to vehicle and pamidronate injections; c) CCl_4_+V = mice subjected to CCl_4_ and vehicle injections; and d) CCl_4_+P = mice subjected to CCl_4_ and pamidronate injections. CCl_4_ or vehicle was administered for 8 weeks, while pamidronate or vehicle was injected at the end of the fourth week. Bone histomorphometry and biomechanical analysis were performed in tibiae, while femora were used for micro-computed tomography and gene expression.

**RESULTS:**

CCl_4_ mice exhibited decreased bone volume/trabecular volume and trabecular numbers, as well as increased trabecular separation, as determined by bone histomorphometry and micro-computed tomography, but these changes were not detected in the group treated with pamidronate. CCl_4_ mice showed increased numbers of osteoclasts and resorption surface. High serum levels of receptor activator of nuclear factor-κB ligand and the increased expression of tartrate-resistant acid phosphatase in the bones of CCl_4_ mice supported the enhancement of bone resorption in these mice.

**CONCLUSION:**

Taken together, these results suggest that bone resorption is the main mechanism of bone loss in chronic hepatocellular disease in mice.

## INTRODUCTION

The liver is a multifunctional organ that occupies a key position in the modulation of carbohydrate, protein, and lipid metabolism. At the same time, it plays a critical role in mineral metabolism and growth. As expected, chronic hepatic disorders are included in the list of diseases considered to be potential causes of osteoporosis 1. Hepatic osteodystrophy (HOD) is the generic name for the heterogeneous bone diseases associated with chronic liver disease, and, HOD combines components of osteoporosis and osteomalacia 2, 3.

In the literature, there has been a convergence of evidence suggesting that bone loss is the primordial bone disorder during the early stages of hepatic disease 4. Vitamin D deficiency appears later, with osteomalacia being an advanced manifestation 5. Disagreement persists regarding the pattern of bone loss. Some studies have shown that decreased bone formation is the predominant impairment in bone remodeling 6, while others have shown that increased bone resorption is the main cause of bone loss in HOD 7. Moreover, cholestasis could be an additional component that may contribute to the differences between previously obtained results 8, 9.

Cross-sectional 10 and longitudinal 11 clinical studies have shown that cholestasis impairs bone mass development in children and adolescents with non-severe cholestatic disease. In these studies, the serum levels of endocrine IGF-I were reduced. The importance of endocrine and autocrine/paracrine IGF-I, as well as reduced bone formation, for the generation of osteopenia in cholestasis was also emphasized in rats subjected to bile duct ligation 12. In addition to the reduction of bone volume (BV/TV), these animals exhibited low serum levels of IGF-I and decreased expression of growth hormone receptors and IGF-I in bone 12. In these studies, there were no indications of increased bone turnover or osteoclast activity, but a lower rate of mineral apposition indicated low osteoblastic activity 12.

Bisphosphonates, including disodium pamidronate, are analogs of inorganic pyrophosphates that exhibit carbon linked to phosphate (P-C-P) instead of to oxygen. The main pharmacological effect of bisphosphonates is that they inhibit bone resorption. Previous experimental and clinical studies have shown the benefits of pamidronate treatment in HOD 2.

The present study was designed to evaluate the mechanisms involved in the emergence of HOD in non-cholestatic liver disease. A pharmacological model of cirrhosis was used, with C57BL/6 mice subjected to the administration of carbon tetrachloride (CCl_4_). The effects of disodium pamidronate on bone were also evaluated. Bone remodeling and microstructure were assessed by histomorphometry in tibiae and by micro-computed tomography (micro-CT) in femora, respectively. There are no data in the literature concerning the expression of genes directly or indirectly involved in bone formation and bone resorption in the bones of cirrhotic mice. Therefore, the final target of the present study was to evaluate the gene expression of RUNX-2, receptor activator of nuclear factor-κB ligand (RANKL), osteoprotegerin (OPG) (Applied Biosystems, Foster City, CA, USA), and tartrate-resistant acid phosphatase (TRAP) in the bones of control and cirrhotic mice submitted or not to disodium pamidronate therapy.

## MATERIALS AND METHODS

The experimental protocol was approved by the Institutional Animal Care and Use Committee of the Ribeirão Preto Medical School, University of São Paulo (protocol no. 111/2011). The animal experiments were performed in strict accordance with international guidelines, as recommended in the Guide for the Care and Use of Laboratory Animals of the Ribeirão Preto Medical School, USP, Animal Research Committee. The mice were divided into 4 groups of 15 animals each as follows: a) C = mice received intraperitoneal (IP) injections of vehicle for 8 weeks and one IP injection of saline solution at the end of the fourth week; b) C+P = mice received IP injections of vehicle and one IP injection of disodium pamidronate; c) CCl_4_ = mice received IP injections of CCl_4_ for 8 weeks and one IP injection of saline solution; and d) CCl_4_+P = mice received, twice per week, IP injections of CCl_4_ for 8 weeks and one IP injection of disodium pamidronate at the end of the fourth week. The clinical conditions of the animals were routinely evaluated once per day in the morning to avoid unnecessary additional stress. There was a mortality rate of 25% in the groups of mice subjected to the IP injection of CCl_4_, and this rate was not influenced by the additional administration of disodium pamidronate.

The central animal facility of the Ribeirão Preto Medical School, USP, provided all of the animals used during the study. Five-week-old male mice weighing approximately 18 g were housed in cages in a room with controlled humidity and temperature (23±1°C) conditions and with an artificial light/dark cycle of 12 hours (lights on: 06:00 AM-06:00 PM). The animals had free access to tap water and pellet chow.

To induce the cirrhosis model, the mice were treated with CCl_4_ (1 mL/kg body weight) dissolved in olive oil 1:4 (v:v) administered by intraperitoneal injection twice per week, as adapted from previous studies in this line of investigation 13, 14. At the end of the 8-week period of CCl_4_ injection, a two-week interval was allowed to elapse to allow for the consolidation of hepatic disease. Mice in the C+P and CCl_4_+P groups were treated with an IP injection of disodium pamidronate (Eurofarma, Ribeirão Preto, SP, Brazil) dissolved in 0.9% NaCl at 1.25 mg/kg at the end of the fourth week 15. At the end of the tenth week, the mice were sacrificed by cervical dislocation, and the blood was immediately collected.

### Biochemical assessment

Serum samples were stored after the end of the experimental protocol for future determinations. Aspartate transaminase (AST) and alanine transaminase (ALT) activities were measured by colorimetry (Labtest, Lagoa Santa, MG, Brazil) with a spectrophotometer (SpectraMax M-5, Molecular Devices, Biocompare, San Francisco, CA, USA). RANKL and OPG were determined by ELISA, as indicated by the manufacturer (R&D Systems, USA). The intra-assay coefficients of variation were 7.2% and 5.3% for RANKL and OPG, respectively.

### Image evaluation

The left femora were scanned using a micro-CT instrument (1172; SkyScan, Kontisch, Belgium), as previously described 16. The trabecular bone volume fraction and microarchitecture of the secondary spongiosa were assessed in the distal portion of the right femora starting at 0.25 mm proximal to the distal growth plate and covering proximally a total length of 1 mm. The bones were scanned at low resolution, with an energy level of 55 kVp and intensity of 145 μA. The equipment underwent a weekly programmed calibration with phantoms presenting with densities of 0.25 and 0.75 mg/cm^3^. The CT-analyzer software, version 1.13.2.1, was used for quantitative assessment. The results are expressed according to standardized nomenclature 17.

### Bone histomorphometry

Quantitative static histomorphometry was performed as previously described 12. In brief, the tibiae were removed and dehydrated in ethanol, infiltrated, and embedded in methyl methacrylate without demineralization. Undecalcified sections were cut at a thickness of 5 μm and were stained with 0.1% toluidine blue. Histomorphometric assessment was performed on the secondary spongiosa of the proximal tibia metaphysis using an OsteoMeasure morphometry system (Osteometrics, Atlanta, GA, USA). The histomorphometric indexes were as follows: bone volume (BV/TV, %), osteoid volume (OV/BV, %), osteoblast surface (Ob.S/BS, %), osteoclast surface (Oc.S/BS), osteoid surface (OS/BS, %), eroded surface (ES/BS, %), trabecular separation (Tb.Sp, μm), trabecular number (Tb.N, /mm), and trabecular thickness (Tb.Th, μm).

### RNA isolation and quantitative real-time PCR

Total femoral RNA was prepared using a standard TRIzol (Sigma-Aldrich) method, according to the manufacturer's instructions. RNA quantity and purity were assessed using a nanophotometer, and a sample with an A260/A280 ratio of 1.9 - 2.1 was used in the present study. cDNA was generated from 1 μg of total RNA using a reverse transcriptase kit (Pre-Improm II, Promega, Madison, WI, USA), according to the manufacturer's instructions. Subsequently, cDNA was used to assess mRNA expression using the TaqMan Fast Advanced Master Mix kit (Applied Biosystems, Foster City, CA, USA) with a thermal cycler StepOnePlus™ Real Time PCR System (Applied Biosystems, Foster City, CA, USA). Relative mRNA expression was determined after normalization for β-actin. RANKL (Mm00441906), OPG (Mm01205928), RUNX-2 (Mm00501584_m1), and TRAP (Mm00475698_m1) inventoried primers were purchased from Applied Biosystems. The expression of target genes was determined by the threshold cycle (CT) values of each sample, which were normalized to the housekeeping genes (2^-ΔCT^).

### Biomechanical testing

Frozen left tibiae were thawed at room temperature for 2 h before testing. Mechanical resistance was assessed on intact tibiae using a destructive three-point bending procedure. Bones were positioned supine on two round bars separated by a distance of 15 mm in a mechanical testing machine (10,000 N, EMIC DL, São José dos Pinhais, PR, Brazil) and were deflected by a notched bar on the opposite side of the bone. The descending speed of the notched bar was 1 mm/min. Maximal force was determined from force/deflection plots.

### Statistical analysis

The results obtained for the four groups were compared using a Kruskal-Wallis one-way analysis of variance followed by Dunn's post-test. Additionally, Spearman's test was used to determine the correlation between two parameters. The statistical analysis was performed using GraphPad Instat software, version 5.0. Differences were accepted as significant at *p<*0.05.

## RESULTS

Body weight gain and mouse final weight were not significantly different between the groups (Table 1). In addition, the liver weights of the CCl_4_ and CCl_4_+P groups were significantly higher than those of the C and C+P groups (*p<*0.05; Table 1).

All of the mice injected with CCl_4_ developed hepatic cirrhosis, which was confirmed by the structural derangements of liver parenchyma in the anatomopathological exam (data not shown), as well as by biochemical changes (Table 1). The mice in the CCl_4_ and CCl_4_+P groups showed higher serum levels of ALT and AST than the other two groups (Table 1). In addition, there was no difference in serum levels of ALT or AST between the two group pairs, i.e., C *vs* C+P and CCl_4_ *vs* CCl_4_+P.

The serum levels of RANKL were significantly higher in the CCl_4_ group (0.087±0.01 pg/mL) than in the C group (0.059±0.02 pg/mL) (*p<*0.05). The cirrhotic group treated with pamidronate (CCl_4_+P = 0.047±0.01) showed serum levels of RANKL similar to those in the control group. The circulatory rates of OPG in the four groups were not significantly different (C = 9.88±4.6 *vs* C+P = 6.2±3.0 *vs* CCl_4_ = 7.52±2.7 *vs* CCl_4_+P = 6.0±3.7 pg/mL).

Table 2 shows the structural characteristics of femoral trabecular bone as evaluated by μCT from the four groups of mice. The CCl_4_ group exhibited lower BV/TV and Tb.N values and higher Tb.Sp values than the C+P group. The CCl_4_+P group showed improved BV/TV, Tb.N, and Tb.Sp values compared with the CCl_4_ group. The trabecular microstructure of the CCl_4_+P group did not differ from that of the C and C+P groups.

Consistent with the results obtained with femoral micro-CT, a bone histomorphometric assessment of the tibiae showed lower values of BV/TV and Tb.N and higher values of Tb.Sp in the CCl_4_ group than in the C+P group (Table 3). In addition, the CCl_4_ group exhibited higher Oc.S/BS and ES/BS values than did the C+P group, whereas the CCl_4_+P group showed lower Oc.S/BS and ES/BS values than the CCl_4_ group.

**Figure 1:**
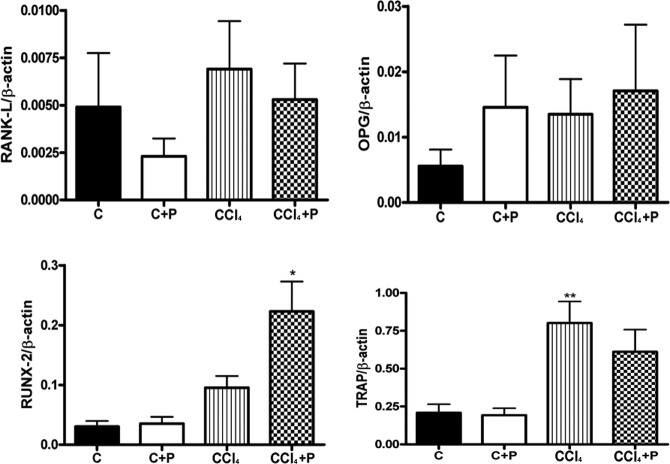
Bone mRNA levels of RANKL, osteoprotegerin, RUNX-2, and TRAP in the four groups (C, C+P, CCl4, and CCl_4_+P). All expression levels are presented relative to β-actin expression (RT-PCR). N=6 per genotype. * CCl_4_+P > C, C+P, and CCl_4_; ** CCl_4_ > C and C+P, *p*<0.05.

In bone, the expression of RANKL and OPG, as well as the RANKL/OPG ratio, was not significantly higher in the CCl_4_ group than in the C group. The expression of TRAP was higher in the CCl_4_ group than in the C and C+P groups. The expression of RUNX-2 was significantly higher in bone from the CCl_4_+P group than in bone from the other three groups (Figure 1).

**Figure 2:**
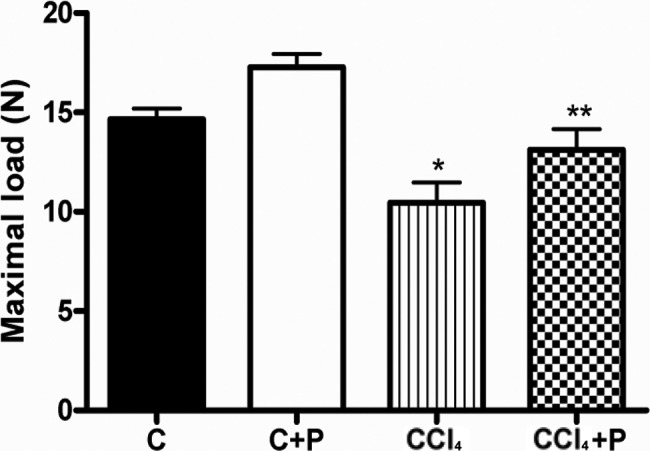
Maximum force attained in the four groups (C, C+P, CCl_4_, and CCl_4_+P). * CCl_4_ < C, C+P, and ** CCl_4_+P < C+P, *p*<0.05.

The force needed to fracture the tibiae was significantly lower in the CCl_4_ group (10.5±3.2 N) than in the C group (14.7±1.7 N) (*p<*0.05; Figure 1). The figure also shows that pamidronate treatment efficiently improved bone resistance in cirrhotic mice. The force needed to fracture bone in the CCl_4_+P group (13.1±3.2 N) was similar to that in the C group but was lower than that in the C+P group (17.3±2.1 N) (Figure 2).

## DISCUSSION

Bone remodeling is a crucial process that enables the skeleton to reconcile two apparently paradoxical properties, i.e., lightness and strength. The sequential activities of bone resorption and formation, when limited to areas with preexisting micro-damage, allow the hard tissue to renew itself, preventing the emergence of bone fragility. The equilibrium of these interdependent activities is complex, involving a close interaction of genetic, nutritional, hormonal, neural, and environmental factors. A deleterious imbalance can result from the excessive activation of osteoclasts or impairment of osteoblast activity. Disease- and drug-induced osteoporosis are associated with one of these two mechanisms or their combination. For example, in glucocorticoid-induced osteoporosis, two distinct phases were identified (18). In the first phase, increased osteoclast resorption prevails, whereas after six months, osteoblast and osteocyte dysfunction predominates. Therefore, in heterogeneous diseases, it can be presumed that either mechanism may exist, depending on the circumstances. Corroborating previous studies that showed the impact of chronic hepatic disorder on the skeleton, the present study contributes to advancements in this line of investigation. Based on the previous experience of our group in the study of bone disturbances in cholestasis (clinical and experimental) 10-12,19, we observed conspicuous differences in bone remodeling using an experimental model of cirrhosis. While the predominant bone impairment seems to be low osteoblastic activity in cholestasis, in hepatocellular disease, increased osteoclastic action appears to be the major mechanism of bone loss. This hypothesis was substantiated by histomorphometric analysis (i.e., increased Oc.S/BS in the CCl_4_ group, while there was no difference in Ob.S/BS between groups) and by biochemical markers of bone remodeling and gene expression in bone.

Previous studies have emphasized that osteoporosis is a major complication of chronic hepatic diseases. Additionally, it is well accepted that the prevalence and severity of bone loss are increased in cholestasis 2. In a previous cross-sectional study, we demonstrated that children with congenital cholestasis had low bone mass 10. In another longitudinal study, we documented impairment of bone mass acquisition 11. In these studies, the serum levels of PTH and 25-hydroxyvitamin D were normal, whereas decreased IGF-I was an early biochemical change in cholestasis 10, 11. Bone loss was an early event in hepatic cholestasis, as 85% of the children were classified as Child-Pugh A and the other 15% were classified as Child-Pugh B. After 3 years, more consistent incremental increases in the serum levels of osteocalcin were observed in the control group (76%) than in children with cholestasis (31%) 11. Moreover, Klein et al. (2002) reported decreased serum levels of osteocalcin and type I collagen telopeptide (ICTP) in children showing advanced cholestatic liver disease 6. Disturbances in bone formation were also observed in rats subjected to bile duct ligation 12. In contrast, in a group predominantly consisting of patients with hepatic liver disease, Crosbie et al. found that bone resorption was the most important cause of bone loss in hepatic osteodystrophy 5. Based on the present findings and previous reports, we hypothesize that cholestatic and hepatocytic disease leads to bone loss by diverse mechanisms. While the impairment of bone formation is the hallmark of cholestatic disorders, bone resorption prevails in hepatocytic diseases. Nevertheless, both disorders are associated with osteoporosis. In the present study, we detected decreased bone volume and trabecular number, as well as increased trabecular separation, in the tibiae and femora of mice with cirrhosis induced by CCl_4_. These results agree with those recently obtained by Naussler et al. (2014) 20. In addition, the present study demonstrated bone weakness by biomechanical tests and showed that pamidronate efficiently reversed bone loss and improved bone strength. The present study reinforced the positive effect of disodium pamidronate on the skeleton, improving bone mass as well as bone strength. It should be emphasized that disodium pamidronate, an antiresorptive drug, not only increased bone mass in diseases associated with increased bone resorption but also improved bone mass in disorders characterized by low bone formation.

RANKL, its cellular receptor RANK, and the decoy receptor OPG are cytokines involved in the crosstalk between osteoblasts and osteoclasts. RANKL produced by osteoblasts dictates osteoclast differentiation and activity through RANK activation in the membranes of osteoclast precursors. OPG produced by osteoblasts deactivates RANKL, thus hampering osteoclastogenesis. In mice subjected to CCl_4_ injection, the serum levels of RANKL were higher than those in the control group. Additionally, pamidronate decreased the circulatory levels of RANKL. These results are consistent with data obtained in the ATP-binding cassette transporter B4 knockout mouse (*Abcb4^−/−^*), which is a well-established experimental model of hepatic disease. In addition to the compatible results regarding bone phenotypes obtained by micro-CT analysis, the present study also revealed similar results related to the serum levels of RANKL 21. In addition, our results showed that cirrhotic mice treated with pamidronate had significantly lower serum levels of RANKL. Moreover, we showed that RANKL expression in bone was slightly increased in mice with hepatic osteodystrophy. The increased expression of TRAP in bone was additional evidence of enhanced osteoclast action in the bones of mice exhibiting hepatic osteodystrophy. To the best of our knowledge, the present study is the first to evaluate TRAP, a marker of osteoclast expression, in bones from cirrhotic mice. The increased expression of TRAP in the CCl_4_ group was consistent with the higher numbers of osteoclasts on the trabecular surface and the deteriorated bone structure of the same group. Moreover, these results indirectly agreed with a previous study showing higher levels of biochemical markers of bone resorption in the serum of cirrhotic individuals, particularly in those with synthetic dysfunction 22.

Previous studies have shown contradictory results related to the osteogenic effects of bisphosphonates. Bisphosphonates are antiresorptive drugs; their metabolic and pro-apoptotic effects strongly hamper osteoclast activity. However, their effects on cells of osteoblastic lineages are far less well known. There have been studies showing that bisphosphonates lead to osteoblast apoptosis, inhibiting their differentiation 23. In contrast, there has been evidence indicating that bisphosphonates induce osteoblastic proliferation and differentiation 24-26. Furthermore, there are also data showing that the effects of bisphosphonates on osteoblastogenesis are dose-dependent 26. It was recently verified that bisphosphonate treatment shrinks bone marrow adipose tissue. These data indirectly suggested a preferential commitment of progenitor cells to differentiate into osteoblasts instead of adipocytes 27. Previous *in vitro* studies have shown that other bisphosphonates (i.e., zoledronate, ibandronate, and clodronate) increased the expression of osteogenic genes, including distal-less homeobox 5 (dlx5), RUNX2, the homeobox analogs known as melanocyte-stimulating hormone (MSH) homeobox 1 and 2 (msx1 and msx2), and OCN 28. Taken together, these data agree with our results showing a significant increase in the bone expression of RUNX2, a key transcription factor for osteoblast differentiation, in cirrhotic mice treated with pamidronate. However, after disodium pamidronate treatment, the significant increase in the expression of RUNX2 in bone was restricted to animals treated with CCl_4_. There is no clear explanation for this occurrence, but in a previous study, it was observed that RUNX-2 expression was elevated in the livers of cirrhotic mice previously treated with CCl_4_ 29. The study called attention to RUNX-2 regulating collagen synthesis, demonstrating that myofibroblasts, including hepatic stellate cells and portal fibroblasts, express RUNX-2 29. This insightful study indicated that CCl_4_ could up-regulate RUNX-2 in mesenchymal cells, unlike in osteoblasts 29. No previous study has evaluated the effects of disodium pamidronate in mice previously indirectly subjected to CCl_4_ by injection. The present study encourages further studies to address the mechanism of action of disodium pamidronate in HOD.

This study has some limitations. The dynamic parameters of bone histomorphometry were not evaluated. However, the present findings are important preliminary data for a more comprehensive study examining alterations in bone remodeling, namely, increased bone resorption as the main mechanism of bone loss in cirrhosis predominantly due to hepatocyte disorders.

The present study showed that liver disease provoked by CCl_4_-induced hepatocyte damage profoundly affected bone microstructure and strength. Biochemical, molecular, and histomorphometric evidence suggested that increased bone resorption is the main mechanism involved in the process of bone loss. Based on previous data obtained in our laboratory, these results strikingly differed from bone disturbances associated with hepatic cholestasis, which mainly presents as changes in bone formation.

## AUTHOR CONTRIBUTIONS

de Paula FJ was responsible for the research proposal and the design of the study, discussed all of the results, and wrote the manuscript. Spirlandeli AL participated in the design of the study, collection of all of the data, analysis of the results, and preparation of the manuscript. Dick-de-Paula I, Zamarioli A, Jorgetti V, Ramalho LN, Nogueira-Barbosa MH, Volpon JB, Jord�o AA, Cunha FQ, and Fukada SY participated in the design of the study and the collection of the data and contributed to the preparation of the manuscript.

## Figures and Tables

**Table 1:** Clinical and biochemical phenotypes.

	C	C+P	CCl_4_	CCl_4_+P
Weight (g)	29.0±1.4	28.7±3.5	29.1±1.7	26.7±2.8
Liver weight (mg/g)	51.3±4.6	47.8±3.0	56.4±5.4*	59.6±3.6**
ALT (U/L)	47.9±7.2	43.3±27.8	137.7±33.3*	133.9±22.9**
AST (U/L)	32.3±16.2	36.9±37.3	155.8±19.6*	168.8±17.8**
RANKL(pg/mL)	0.059±0.02	0.061±0.02	0.087±0.01+	0.047±0.01++
OPG (pg/mL)	9.88±4.6	6.2±3.0	7.52±2.7	6.0±3.7

* > C and C+P;

** > C and C+P;

+ > C;

++ < CCl_4_;

*p<*0.05

ALT: alanine transaminase; AST: aspartate transaminase; RANKL: receptor activator of nuclear factor-κB ligand; and OPG: osteoprotegerin.

**Table 2:** Micro-CT assessment in the femora of the four groups.

	C (n=6)	C + P (n=6)	CCl_4_ (n=6)	CCl_4_ + P (n=6)
BV/TV (%)	11.96±3.47	17.22±3.07	10.40±3.53*	12.89±4.44
Tb.Th (mm)	0.054±0.009	0.053±0.002	0.054±0.008	0.047±0.005
Tb.N (1/mm)	2.28±0.28	3.24±0.54	1.67±0.13*	2.25±0.70
Tb.Sp (mm)	0.17±0.02	0.089±0.009+	0.22±0.02**	0.10±0.01++

* < C+P;

** > C+P;

+ < C;

++ < CCl_4_;

*p<*0.05

**Table 3:** Bone histomorphometry in the tibiae of the four groups.

	C (n=7)	C + P (n=6)	CCl_4_ (n=7)	CCl_4_ + P (n=7)
**BV/TV (%)**	10.4±2.9	12.6±2.1	7.9±1.4*	10.6±3.4
**OV/BV (%)**	1.84±1.42	2.39±0.42	1.32±0.77	2.74±1.46
**Tb.Th (µm)**	49.0±5.3	54.3±9.1	56.7±16.6	46.8±9.8
**Tb.N (/mm)**	2.1±0.3	2.3±0.3	1.4±02*	2.2±0.3+
**Tb.Sp (µm)**	439.1±95.4	376.9±58.3	657.7±149.7**	410±80.2++
**Oc.S/BS (%)**	1.4±0.9	0.2±0.3	2.8±2.2**	0.5±0.6++
**ES/BS (%)**	1.6±0.6	0.3±0.4	4.6±2.4**	0.9±0.7++
**Ob.S/BS (%)**	7.3±3.4	8.4±2.6	7.0±4.6	7.7±3.1
**OS/BS (%)**	18.9±9.7	23.08±6.5	14.79±5.2	19.4±7.1

* < C+P;

** > C+P;

+ > CCl_4_;

++ < CCl_4_;

*p<*0.05
